# Common neural structures activated by epidural and transcutaneous lumbar spinal cord stimulation: Elicitation of posterior root-muscle reflexes

**DOI:** 10.1371/journal.pone.0192013

**Published:** 2018-01-30

**Authors:** Ursula S. Hofstoetter, Brigitta Freundl, Heinrich Binder, Karen Minassian

**Affiliations:** 1 Center for Medical Physics and Biomedical Engineering, Medical University Vienna, Vienna, Austria; 2 Neurological Center, Maria Theresien Schloessel, Otto Wagner Hospital, Vienna, Austria; 3 Center for Neuroprosthetics and Brain Mind Institute, School of Life Sciences, Swiss Federal Institute of Technology (EPFL), Lausanne, Switzerland; UniversitatsKlinikum Heidelberg, GERMANY

## Abstract

Epidural electrical stimulation of the lumbar spinal cord is currently regaining momentum as a neuromodulation intervention in spinal cord injury (SCI) to modify dysregulated sensorimotor functions and augment residual motor capacity. There is ample evidence that it engages spinal circuits through the electrical stimulation of large-to-medium diameter afferent fibers within lumbar and upper sacral posterior roots. Recent pilot studies suggested that the surface electrode-based method of transcutaneous spinal cord stimulation (SCS) may produce similar neuromodulatory effects as caused by epidural SCS. Neurophysiological and computer modeling studies proposed that this noninvasive technique stimulates posterior-root fibers as well, likely activating similar input structures to the spinal cord as epidural stimulation. Here, we add a yet missing piece of evidence substantiating this assumption. We conducted in-depth analyses and direct comparisons of the electromyographic (EMG) characteristics of short-latency responses in multiple leg muscles to both stimulation techniques derived from ten individuals with SCI each. Post-activation depression of responses evoked by paired pulses applied either epidurally or transcutaneously confirmed the reflex nature of the responses. The muscle responses to both techniques had the same latencies, EMG peak-to-peak amplitudes, and waveforms, except for smaller responses with shorter onset latencies in the triceps surae muscle group and shorter offsets of the responses in the biceps femoris muscle during epidural stimulation. Responses obtained in three subjects tested with both methods at different time points had near-identical waveforms per muscle group as well as same onset latencies. The present results strongly corroborate the activation of common neural input structures to the lumbar spinal cord—predominantly primary afferent fibers within multiple posterior roots—by both techniques and add to unraveling the basic mechanisms underlying electrical SCS.

## Introduction

Epidural spinal cord stimulation (SCS) is generally known as a neuromodulatory therapy for the relief of chronic, intractable pain of the trunk and limbs [1–3]. Yet it has also a long history of applications in various motor disorders [4–12]. Currently, SCS has experienced a resurgence of interest in spinal cord injury (SCI) research and neurorehabilitation following promising studies that demonstrated the alleviation of severe lower-limb spasticity [13], the generation of motor output underlying lower-limb extension [14] and standing [15,16], the generation [17–19] or augmentation [15,20–22] of rhythmic and locomotor-like lower-limb activity, and specifically, studies that rediscovered the effect of enabling some volitional movements [23] in otherwise paralyzed legs [12,15,24–26] when the stimulation was applied over the lumbar spinal cord.

Computational [27,28] and electromyography (EMG) based [18,29,30] studies in individuals with SCI proposed that epidural lumbar SCS predominantly recruits large-to-medium diameter proprioceptive and cutaneous afferents within lumbar and upper sacral posterior rootlets/roots. The prevailing view is that, depending on the applied SCS parameters [12,30,31], the stimulation-induced afferent input then transsynaptically recruits various spinal reflex circuits [12,29,32], circuits involved in the regulation of proprioceptive input and motoneuronal excitability [13,33], and rhythm and pattern generating neural networks [19,30,34]. This notion was supported by more direct in vivo physiological and pharmacological rat experiments, which confirmed that epidural SCS engages spinal circuits indirectly through the electrical stimulation of afferent root fibers [35,36]. Furthermore, a recent functional neuroanatomical study of the swine lumbar spinal cord demonstrated that also in a large animal model with closer similarity to human vertebral morphometry, an epidural electrode position in proximity to the roots is a critical factor to evoke responses in the hindlimb muscles [37].

The understanding that the motor effects of epidural lumbar SCS are initiated through the recruitment of posterior-root fibers—rather than through the direct electrical stimulation of spinal gray matter neurons—together with earlier observations that transspinal stimulation using surface electrodes could elicit root-evoked short-latency spinal reflexes in the soleus muscle [38,39], motivated the development of transcutaneous SCS [40]. Transcutaneous SCS uses paravertebral and abdominal skin electrodes placed in order to generate a current flow through the lower trunk, directed largely posteroanteriorly and perpendicularly to the spine at the level of the thoracolumbar junction [28,40,41]. The stimulation of posterior-root afferents by this noninvasive method was supported by computer modeling [28,42] and, neurophysiologically, by demonstrating the reflex nature of the evoked muscle responses by the presence of post-activation depression when tested by paired stimuli [40,43,44], their suppression by tendon vibration [40], their modulation by passive and active leg movements [40,45,46], and the increase in their latencies when moving the stimulating electrodes from the level of the lumbar spinal cord to that of the cauda equina [40]. Recent proof-of-concept studies have suggested that this noninvasive method may produce similar neuromodulatory effects in individuals with SCI as epidural SCS, including the attenuation of spinal spasticity [47,48], the augmentation and generation of rhythmic activity during robotic-driven treadmill stepping in individuals with complete SCI [49], the facilitation of residual stepping capability in patients with motor- and sensory-incomplete SCI [43,50,51], as well as the enabling of some voluntary control after paralysis [52,53].

The interpretation that transcutaneous and epidural lumbar SCS both act predominantly through electrical activation of posterior-root afferents was inferred from independent observations of various studies. The aim of the present study was to add a yet missing piece of evidence corroborating the activation of common neural input structures to the spinal cord, based on the hypothesis that both methods would elicit equivalent lower-limb muscle responses. We directly compared the associated evoked EMG potentials in a statistically sound population, the largest of the recent publications of SCS in SCI. The surgical placement of epidural leads with respect to the lumbar spinal cord segments is normally tested by the elicitation of short-latency reflexes in L2–S2 innervated leg muscle groups, which in turn give information about the specifically stimulated roots [13,18,29,30]. According to their site of initiation within the posterior roots and recording from lower-limb muscle groups, these responses were termed posterior root-muscle (PRM) reflexes [18,30]. Likewise, the placement of the paravertebral stimulating surface electrodes in transcutaneous SCS over the lumbar spinal cord is verified based on the elicitation of short-latency responses in the legs [40,43,47,51]. Here, we applied a detailed statistical analysis to demonstrate similarities of the EMG characteristics of such responses in multiple lower-limb muscles between epidural and transcutaneous SCS in 10 individuals with SCI each. Three of these subjects were tested with both techniques at different time points. Therefore, this study shall strengthen the conceptual framework and understanding of the neurophysiological mechanisms underlying electrical stimulation of the human lumbar spinal cord.

## Materials and methods

### Subjects

Data derived from 17 adults with traumatic, chronic (≥ 12 months post-injury) SCI were analyzed. The subjects’ neurological status (Table 1) was evaluated according to the International Standards for Neurological Classification of Spinal Cord Injury [54]. Data were collected in a previous study of the effects of transcutaneous SCS on lower-limb spasticity (subjects 1–10), and as part of the routine clinical evaluation conducted at the Neurological Center, Otto-Wagner-Hospital, Vienna, to monitor the efficacy of epidural SCS to control spinal spasticity (subjects 8–17), respectively (*cf*. [13]). Subjects 8–10 had participated in both protocols at different time points with several days (subject 10), or months (subjects 8, 9) between the recordings and with transcutaneous SCS being applied when the subjects had no epidural system implanted. The individuals tested with transcutaneous SCS had a mean age (± SD) of 39.7 ± 20.1 years, ranging from 18 to 70 years, a mean time since injury of 4.5 ± 2.8 years, ranging from 1 to 9 years, and a mean body height of 178.6 ± 10.3 cm, ranging from 158 to 192 cm. Those tested with epidural SCS had a mean age of 26.4 ± 9.7 years, ranging from 18 to 52 years, a mean time since injury of 4.8 ± 2.4 years, ranging from 2 to 9 years, and a mean body height of 179.4 ± 8.1 cm, ranging from 161 to 192 cm. There were no statistical differences between groups in age (Student’s t-test, P = .076), time since injury (P = .796), and body height (P = .849). The subjects of the study of transcutaneous SCS had signed an informed consent form prior to their participation. The clinically obtained data of epidural SCS were retrospectively analyzed. Study protocols and retrospective data analysis were approved by the Ethics Committee of the City of Vienna (EK-11-078-0511, EK-17-059-VK).

**Table 1:** Subjects’ data and neurological status according to the International Standards for Neurological Classification of Spinal Cord Injury.

Subject	Sex	Age (y)	AIS	Neurol. level of injury	Years after injury	Height (cm)	Weight (kg)	Type of SCS
1	M	66	D	T4	7	180	82	t
2	F	53	D	C7	8	170	60	t
3	M	47	D	C4	4	186	81	t
4	F	70	D	T6	2	170	63	t
5	M	21	C	C6	2	190	80	t
6	M	20	C	T6	5	180	57	t
7	F	18	A	T7	1	158	50	t
8	M	52	D	C7	2	178	77	e,t
9	M	26	C	C4	9	192	67	e,t
10	M	24	C	C5	5	182	74	e,t
11	M	29	B	C6	3	181	75	e
12	M	22	A	C6	5	177	63	e
13	M	28	A	C5	6	185	85	e
14	M	18	A	C5	3	180	60	e
15	M	19	A	T2	8	174	70	e
16	M	21	A	T8	3	184	67	e
17	F	25	A	T6	4	161	63	e

AIS, American Spinal Injury Association Impairment Scale; e, epidural; SCS, spinal cord stimulation; t, transcutaneous

### Transcutaneous SCS

Transcutaneous SCS was applied through self-adhesive hydrogel surface electrodes (Schwamedico GmbH, Ehringshausen, Germany), using a pair of interconnected electrodes (each 5 cm in diameter) placed over the T11 and T12 spinous processes, on both sides of the spine, and a pair of interconnected indifferent electrodes (8 x 13 cm each) placed para-umbilically on the lower abdomen [40,47,51]. A current-controlled stimulator (Stimulette r2x+, Dr. Schuhfried Medizintechnik GmbH, Moedling, Austria) delivered charge-balanced, symmetric, biphasic rectangular pulses of 1-ms width per phase. With reference to the indifferent electrodes, the paravertebral electrode pair was the anode for the first and the cathode for the second phase of the biphasic pulse. Paravertebral electrode placement corresponding to the rostrocaudal levels of the lumbosacral spinal cord was verified by applying single stimulation pulses to elicit muscle responses in the L2–S2 innervated rectus femoris (RF), biceps femoris (BF), tibialis anterior (TA), and triceps surae muscle group (TS) with the subjects lying relaxed in the supine position [40,55]. The stimulation amplitude was slowly increased and the response thresholds for the individual muscle groups were documented. The target intensity was the ‘common threshold’ [40], at which responses with peak-to-peak amplitudes ≥ 100 μV in all muscle groups studied were evoked, but stimulation amplitudes were never increased beyond the individually tolerable maximum. Across subjects, this common threshold intensity was 64 ± 19 mA (mean ± SE; per phase of the biphasic stimulation pulse), with a range of 32 to 86 mA. At this stimulation amplitude, three paired pulses, each at an interstimulus interval of 50 ms, were applied. The relative attenuation of the responses elicited by the respective second stimulation pulses was assessed to test the presence of post-activation depression and hence verify the reflex nature of the evoked muscle responses, i.e., the electrical stimulation of sensory fibers [40,41,56,57]. Subsequently and with unchanged stimulation amplitude, another 65 single stimuli were applied with 15 seconds between successive stimuli.

### Epidural SCS

Epidural SCS was applied using percutaneous leads (subjects 9–17, Model 3487A; subject 8, Model 3777; Medtronic, Minneapolis, MN) placed longitudinally in the posterior epidural space over the lumbosacral spinal cord, individually ranging from T11–L1 vertebral levels, and connected to a pulse generator (subjects 9–17, Itrel 3; subject 8, Prime Advance; Medtronic) implanted in a subcutaneous pocket created laterally in the abdominal region. The lead carried either four (subjects 9–17, referred to as 0–3 from rostral to caudal direction) or eight (subject 8, referred to as 0–7) cylindrical electrodes, each having a diameter of 1.3 mm and a length of 3 mm, with an inter-electrode spacing of 6 mm. Each electrode could be set to +,–, or ‘off’, allowing for, e.g., the use of various bipolar electrode combinations. All recordings had been collected with the patients lying relaxed in the supine position. For the present analysis, we considered recordings during epidural SCS with the bipolar electrode combinations of 0–3+ and 0+3–, i.e., with either the most rostral or most caudal contact of the 3487A lead selected as the stimulating cathode (‘–’), as these two settings were routinely tested in all patients. In subject 8, the electrode combinations of 4–7+ and 4+7– (i.e., same spacing between active electrodes as in the other subjects) were used. The pulse generators delivered capacitively decoupled monophasic rectangular constant-voltage pulses of 210 μs width followed by exponentially decaying phases, adjusting charge balance and avoiding delivery of direct current components [27]. The lowest programmable stimulation frequency was 2 Hz, and stimulation amplitudes could be set to 0–10.5 V. Response thresholds for each muscle group were identified for stimulation with the bipolar electrode combinations 0–3+ (4–7+) and 0+3– (4+7–) at 2 Hz. For the analysis of the EMG characteristics of the evoked responses, we used EMG recordings during epidural SCS with both electrode combinations at 2 Hz and with common threshold intensity. The common threshold intensity across subjects was 4.4 ± 0.5 V, with a range of 2–6.5 V. 2-Hz stimulation with a given intensity was normally applied for about 30 s, with actually 60.1 ± 7.8 responses per muscle group and subject available on average, with a range from 29 to 84. To test the degree of post-activation depression, the responses to the first two stimuli of a train at 20 Hz with common threshold intensity were extracted from the recordings obtained with the afore-mentioned bipolar electrode combinations and for each muscle group. The relative attenuation of the responses to the second stimulation pulses applied at an interval of 50 ms was calculated.

### Data acquisition

Surface-EMG activity was recorded from RF, BF, TA, and TS bilaterally with pairs of silver-silver chloride electrodes (Intec Medizintechnik GmbH, Klagenfurt, Austria), placed centrally with a longitudinal alignment and an inter-electrode distance of 3 cm [58]. Specifically, the EMG electrode locations were, for RF, half way on the line from the superior anterior iliac spine and the superior part of the patella; for BF, over the muscle (identified by a brief passive stretch at the knee using the hypertonia of the muscle or by voluntary contraction), between the greater trochanter and the lateral epicondyle of the femur; for TA, at 1/3 on the line between the fibular head and the medial malleolus; and for TS, over the midline between the medial and lateral heads of the gastrocnemius muscle at their distal ends, partially over the soleus (also see Discussion). Common ground electrodes were placed bilaterally over the fibular heads. Stimulation artifacts, required to relate the lower-limb muscle responses to the stimulation pulse that had triggered them, were acquired by an additional pair of surface-EMG electrodes placed unilaterally over the lumbar paraspinal trunk muscles in case of epidural SCS and by the EMG electrodes over the BF for transcutaneous SCS. Abrasive paste (Nuprep, Weaver and Company, Aurora, CO) was used for skin preparation to reduce EMG electrode resistance below 5 kΩ. In subjects 1–9, EMG signals were recorded via instrumentation amplifier (INA 118, Texas Instruments Inc., Dallas, TX), amplified with a gain of 600, filtered to a bandwidth of 10–500 Hz within the analogue front end and digitized at 2048 samples per second and channel with a USB-NI 6261 data acquisition card (National Instruments Inc., Austin, TX) and recorded using DasyLab 11.0 (Measurement Computing Corporation, Norton, MA). In the remaining subjects, EMG signals were amplified (Grass Instruments, Quincy, MA) with a gain of 2000, filtered to a bandwidth of 30–700 Hz, and digitized at 2002 samples per second and channel using a Codas ADC system (Dataq Instruments, Akron, OH). To account for the different high-frequency cutoff settings during data acquisition that could potentially influence, e.g., the initial deflection of the evoked potential from baseline and thus the onset latency, all EMG data were additionally low-pass filtered offline at 500 Hz.

### Data analysis and statistics

Data analysis was performed offline using Matlab R2012a (The MathWorks, Inc., Natick, MA). Statistical analysis was done with IBM SPSS Statistics 21.0 for Windows (IBM Corporation, Armonk, NY). For Student’s t-tests, the assumption of normality was tested using Shapiro-Wilk test and equality of variances was verified using Levene's test. α-errors of P < .05 were considered significant.

To test for the occurrence of post-activation depression, the peak-to-peak amplitudes of the first and the second responses to the double pulses applied through transcutaneous SCS and of the responses to the first two pulses of the 20-Hz trains of epidural SCS were determined. The results obtained with the two epidural bipolar electrode combinations were pooled, because the responses to epidural SCS with the posteriorly positioned electrodes could be expected to be of reflex nature in any case [18,27]. For each stimulation technique, the amplitudes of the responses to the first and second stimulation pulses were compared using a paired Student’s t-test.

For detailed analysis of the evoked potentials, the EMG of the responses in each muscle group to single-pulse transcutaneous and 2-Hz epidural SCS was analyzed for time windows of 50 ms post-stimulus. Average peak-to-peak amplitudes of the responses obtained with the target intensity (common threshold or the maximum tolerable intensity) were calculated per muscle group and subject for transcutaneous SCS and separately for both bipolar epidural electrode combinations. The peak-to-peak amplitudes attained with the two epidural electrode combinations were separately analyzed because we assumed that the 0–3+ combination would more dominantly activate the upper lumbar spinal cord segments while the 0+3– combination would rather activate lower lumbar/upper sacral segments [18,29,30,32]. Group results across subjects (± SE) for each muscle group studied were obtained from the individual mean peak-to-peak amplitudes and separate one-way analyses of variance (ANOVAs) were calculated for each muscle group to test for differences in the peak-to-peak amplitudes that were attainable under the three different stimulation conditions (transcutaneous SCS and epidural SCS with the two bipolar electrode combinations). To further study the segmental selectivity, we documented the response thresholds of each muscle group and subject for transcutaneous and epidural SCS with the two electrode combinations. For better comparability, thresholds were normalized to the lowest value across muscles for a given subject and stimulation condition. These normalized response thresholds of the different muscle groups were then compared separately for each stimulation condition using one-way ANOVAs. Effect sizes of one-way ANOVAs were reported by the partial eta-squared ${(\eta }_{\text{p}}^{2})$. When statistical significant differences were found (P < .05), Bonferroni-corrected Student’s t-tests were calculated.

Onset and offset latencies were calculated for the responses to single-pulse transcutaneous and to 2-Hz epidural SCS and determined as the times between the effective phases of the stimulation pulses and the first and last deflections of the EMG potential from baseline, respectively, that exceeded 5% of the corresponding peak-to-peak amplitudes. For transcutaneous SCS, the onset of the effective phase was at the transition between the two phases of the biphasic stimulation pulses, when the function of the paravertebral electrodes changed from anode to cathode [40] (S1 Fig). This change in polarity was well reflected in the shape of the stimulus artifacts as recorded by the EMG electrodes over the BF, the EMG site located closest to the paravertebral stimulation site. In case of the quasi-monophasic stimulation pulses delivered by epidural SCS, the latencies were measured from the onset of the stimulation artifact. The response durations were defined as the times between onset and offset latencies. In case of epidural SCS, onset and offset latencies as well as durations of the responses obtained with both bipolar electrode combinations were pooled. For each subject and muscle group, mean onset and offset latencies, and durations were then calculated from all available responses of the left and right side. Group values (± SE) for transcutaneous and for epidural SCS were obtained by averaging the corresponding mean values. Mean onset and offset latencies, and durations of the responses to the two stimulation techniques were compared using Student’s t-tests.

Across subjects, mean EMG waveforms of the responses to either technique elicited with the respective common threshold intensities were obtained by stimulus-triggered averaging of the available set of EMG responses per muscle group studied.

Additionally, onset and offset latencies as well as response durations of the responses to transcutaneous and epidural SCS derived from the three subjects tested with both stimulation techniques were compared individually using Student’s t-tests.

## Results

2-Hz epidural and single-pulse transcutaneous SCS applied with the common threshold intensities produced twitch-like contractions in multiple lower-limb muscle groups bilaterally with each stimulation pulse delivered. Associated EMG responses with peak-to-peak amplitudes ≥ 100 μV were evoked in all recorded muscle groups by epidural SCS, and in all muscle groups with the exception of one TA of subject 8 and unilateral TA and TS of subject 7 (declared as missing data) by transcutaneous SCS.

**Fig 1:**
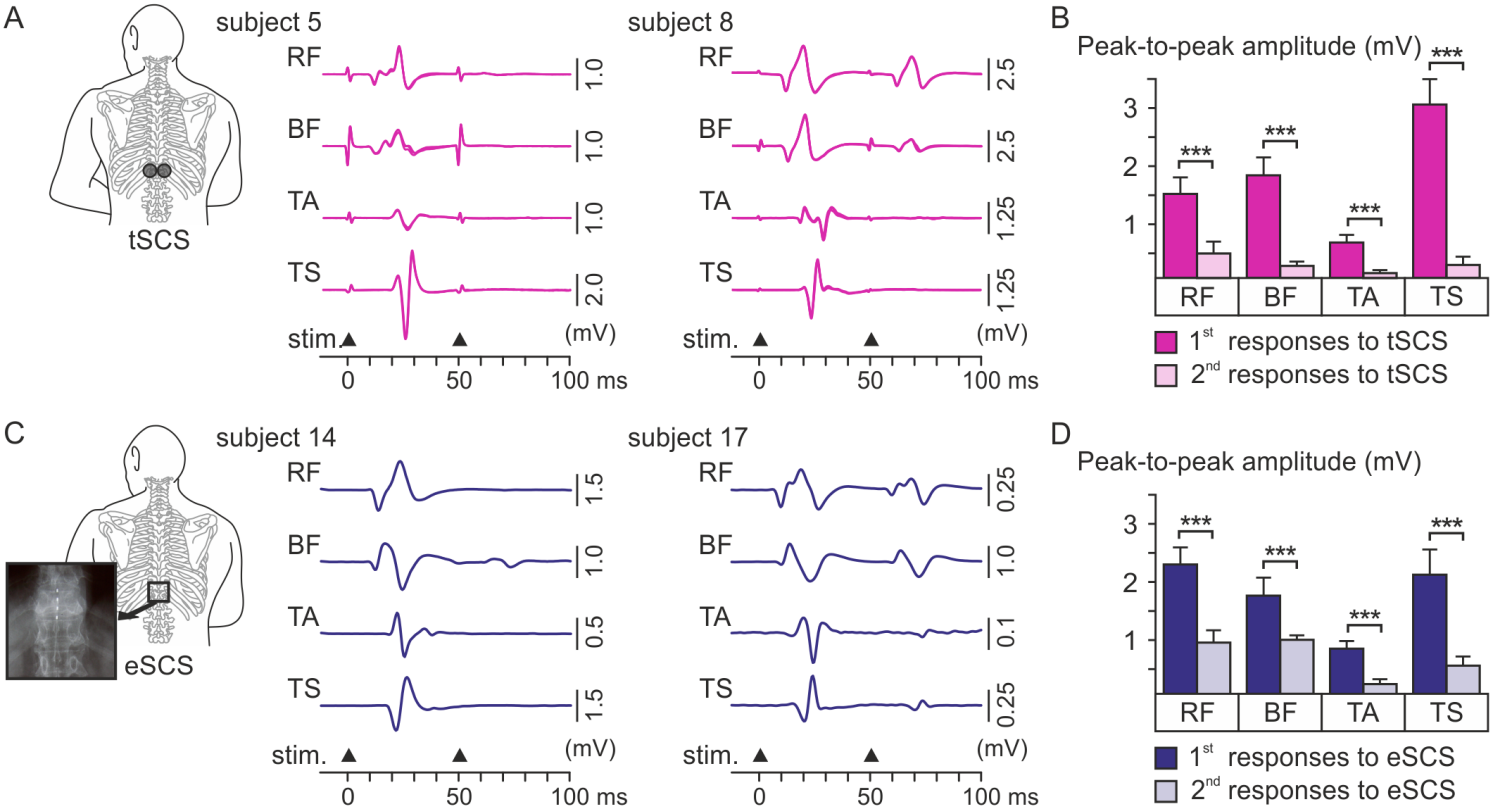
Post-activation depression of evoked EMG responses to transcutaneous and epidural SCS. (A) Exemplary EMG responses to paired stimuli (stim., black triangles) applied transcutaneously with a 50-ms interstimulus interval evoked in rectus femoris (RF), biceps femoris (BF), tibialis anterior (TA), and triceps surae muscle group (TS); responses of one lower limb of subjects 5 and 8. Shown are three repetitions superimposed. The initial brief biphasic peaks are stimulation artifacts consistently generated with the surface-electrode based stimulation. Two examples are shown to illustrate the intraindividual variability of post-activation depression. (B) Group results across subjects tested with transcutaneous SCS (tSCS). (C) Exemplary EMG responses to the first two pulses of a 20-Hz train applied epidurally, subjects 14 and 17. (D) Group results across subjects with epidural SCS (eSCS) considering data obtained with both bipolar electrode combinations tested. Responses to the second pulses (light purple bars in (B) and light blue bars in (D)) were significantly depressed compared to those evoked by the first pulses (purple bars in (B) and blue bars in (D)) for both transcutaneous and epidural SCS. Bars are group means, error bars indicate SE. Asterisks indicate significant effects (***, P < .001).

When paired pulses with an interstimulus interval of 50 ms were applied either epidurally or transcutaneously, the EMG amplitudes of the responses evoked by the second stimulation pulse were significantly decreased in all muscle groups across subjects (Fig 1; paired Student’s t-test: transcutaneous SCS: RF, difference: 1060.3 ± 164.7 μV; ratio: 0.18 ± 0.06; t_19_ = 6.438, P = .000; BF, 1620.3 ± 268.6 μV; 0.12 ± 0.03; t_19_ = 6.033, P = .000; TA, 544.3 ± 117.0 μV; 0.12 ± 0.05; t_18_ = 4.653, P = .000; and TS, 2871.2 ± 410.5 μV; 0.05 ± 0.03; t_18_ = 6.995, P = .000; epidural SCS: RF, difference: 1382.2 ± 253.5 μV; ratio: 0.32 ± 0.07; t_19_ = 5.453, P = .000; BF, 782.3 ± 114.0 μV; 0.53 ± 0.05; t_19_ = 6.862, P = .000; TA, 626.6 ± 118.4 μV; 0.10 ± 0.04; t_19_ = 5.294, P = .000; and TS, 1609.0 ± 254.4 μV; 0.16 ± 0.05; t_19_ = 6.324, P = .000). Between the two stimulation methods, there were no statistical differences in the amplitude ratios of the responses to the paired stimuli for RF (Student’s t-test; P = .120), TA (P = .749), and TS (P = .081), while there was a significant difference in case of BF (P < .001). Overall, the post-activation depression identified the EMG potentials evoked by both stimulation techniques as reflex responses.

**Fig 2:**
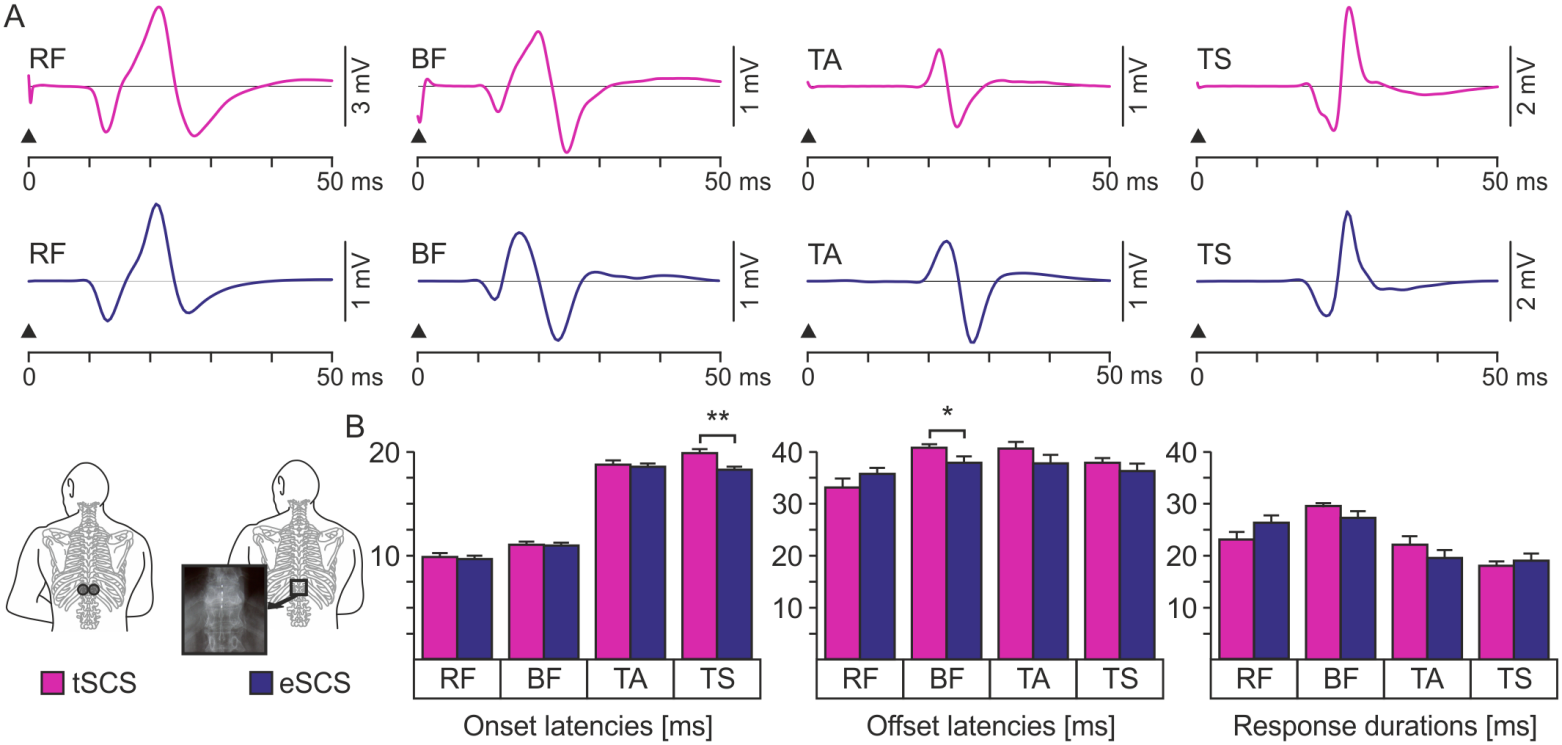
EMG characteristics of responses to transcutaneous and epidural SCS. (A) Characteristic EMG waveforms of responses to single-pulse transcutaneous (tSCS) and 2-Hz epidural SCS (eSCS) with respective common threshold intensities, obtained by stimulus-triggered averaging of the available set of evoked potentials derived from rectus femoris (RF), subjects 8 (transcutaneous stimulation) and 15 (epidural stimulation); biceps femoris (BF), subjects 4 and 13; tibialis anterior (TA), subjects 6 and 16; and the triceps surae muscle group (TS), subjects 9 and 13. The individual examples were selected to illustrate the most representative muscle-specific shapes of the EMG potentials. Black arrowheads indicate the onsets of the effective phases of the stimulation pulses. (B) Group results of onset latencies, offset latencies, and EMG potential durations of the responses to transcutaneous (purple bars) and epidural SCS (blue bars). Bars are group means, error bars indicate SE. Asterisks indicate significant differences (*, P < .05; **, P < .01).

The evoked potentials had muscle-specific bi- or triphasic EMG waveforms that were very much alike for both stimulation techniques (Fig 2A). Mean onset latencies (± SE) of the responses across the two subject groups were RF, 9.8 ± 0.4 ms (transcutaneous SCS) and 9.6 ± 0.3 ms (epidural SCS); BF, 11.0 ± 0.3 ms and 10.9 ± 0.2 ms; TA, 18.7 ± 0.4 ms and 18.5 ± 0.3 ms; and TS, 19.8 ± 0.4 ms and 18.2 ± 0.3 ms (Fig 2B). No statistical differences were found for RF (t_18_ = .476, P = .640), BF (t_18_ = .555, P = .586), and TA, (t_18_ = .275, P = .787). The mean onset latency of TS responses to epidural SCS was significantly shorter compared to that obtained for transcutaneous SCS (t_18_ = 3.212, P = .005). Offsets were RF, 33.2 ± 1.6 ms (transcutaneous SCS) and 35.9 ± 1.2 ms (epidural SCS); BF, 40.7 ± 0.7 ms and 37.8 ± 1.1 ms; TA, 40.5 ± 1.3 ms and 37.7 ± 1.6 ms; and TS, 37.8 ± 0.9 ms and 36.2 ± 1.4 ms. No statistical differences were detected (RF, t_18_ = -1.367, P = .188; TA, t_18_ = 1.363, P = .190; and TS, t_18_ = .944, P = .358) except for the offsets of the responses in the BF (t_18_ = 2.369, P = .029). The durations of the responses to the two stimulation techniques revealed no differences and were RF, 23. 1 ± 1.4 ms (transcutaneous SCS) and 26.3 ± 1.3 ms (epidural SCS; t_18_ = -1.611, P = .125); BF, 29.5 ± 0.8 ms and 27.2 ± 1.1 ms (t_18_ = 1.630, P = .120); TA, 22.0 ± 1.6 ms and 19.5 ± 1.5 ms (t_18_ = 1.150, P = .265), and TS, 18.0 ± 0.8 ms and 19.0 ± 1.4 ms (t_18_ = -.640, P = .530).

**Fig 3:**
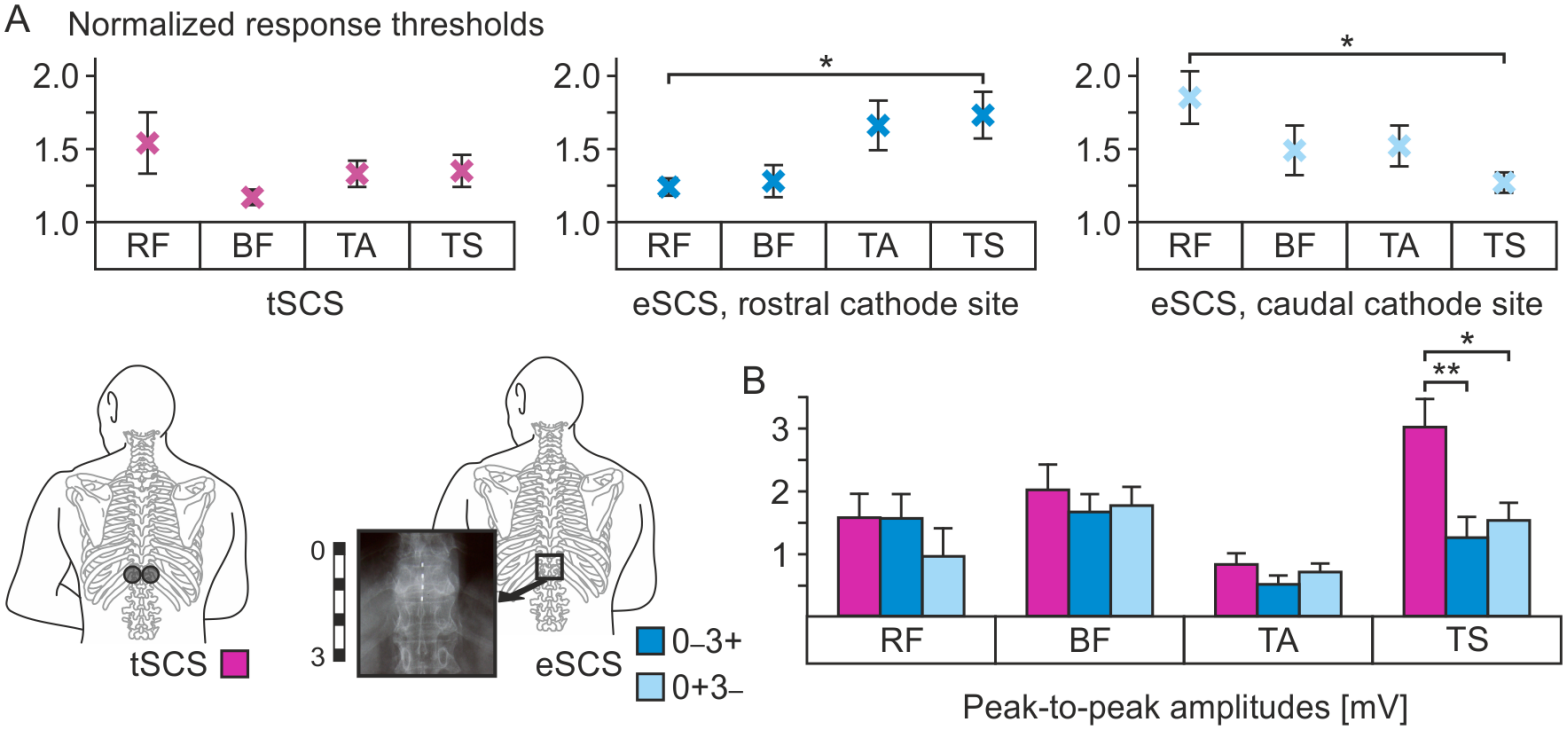
Normalized thresholds of responses to transcutaneous and epidural SCS and peak-to-peak amplitudes at common-threshold intensity. (A) Normalized thresholds for rectus femoris (RF), biceps femoris (BF), tibialis anterior (TA), and the triceps surae muscle group (TS) for transcutaneous SCS (tSCS, purple crosses), epidural SCS (eSCS) with a rostral cathode site (mid-blue crosses), and epidural SCS with a caudal cathode site (light blue crosses). (B) Group results of response sizes of RF, BF, TA, and TS to transcutaneous SCS (purple bars) as well as epidural SCS with a bipolar electrode combination using a rostral cathode site (mid-blue bars) and a caudal cathode site (light blue bars) applied with the respective common threshold intensities. Crosses in (A) and bars in (B) are group means, error bars indicate SE. Significant results of the pairwise post-hoc tests are indicated with asterisks (*, P < .05; **, P < .01).

The mean normalized thresholds (± SE) for eliciting evoked potentials by transcutaneous SCS were RF, 1.54 ± 0.21; BF, 1.17 ± 0.04; TA, 1.33 ± 0.09; and TS, 1.35 ± 0.11; by epidural SCS with rostral cathode site RF, 1.24 ± 0.06; BF, 1.28 ± 0.11; TA, 1.66 ± 0.17; and TS, 1.73 ± 0.16; and by epidural SCS with caudal cathode site RF, 1.85 ± 0.18; BF, 1.49 ± 0.17; TA, 1.52 ± 0.14; and TS 1.27 ± 0.07 (Fig 3A). Between muscle groups, the respective relative response thresholds differed significantly in case of epidural SCS with both cathode locations (rostral cathode location: one-way ANOVA, F_3,76_ = 3.708, P = .015, $\eta_{\text{p}}^{2}=.128;$ caudal cathode location: F_3,76_ = 2.752, P = .048, $\eta_{\text{p}}^{2}=.098$). Post-hoc testing revealed differences between RF and TS thresholds in both cases (rostral cathode location, P = .048; caudal cathode location, P = .029), but not for the other pairwise comparisons (RF vs. BF: rostral cathode location, P = .995, and caudal cathode location, P = .287; RF vs. TA. P = .114, and P = .376; BF vs. TA, P = .186, and P = .998; BF vs. TS, P = .085, and P = .719; and TA vs. TS, P = .982, and P = .613). No differences in relative response thresholds between muscle groups were detected in case of transcutaneous SCS (F_3,73_ = 1.344, P = .267 $\eta_{\text{p}}^{2}=.052$). At the common threshold, the mean peak-to-peak amplitudes (± SE) of the EMG potentials evoked by single-pulse transcutaneous SCS as well as 2-Hz epidural SCS with either of the two bipolar electrode combinations (Fig 3B) showed no statistical differences in case of RF (one-way ANOVA, F_2,27_ = .748, P = .483, $\eta_{\text{p}}^{2}=.053$), BF (F_2,27_ = .301, P = .743, $\eta_{\text{p}}^{2}=.022$), and TA (F_2,27_ = 1.351, P = .276, $\eta_{\text{p}}^{2}=.091$). In TS, the response sizes were significantly different (F_2,27_ = 6.753, P = .004, $\eta_{\text{p}}^{2}=.333$). Post-hoc tests showed significantly larger amplitude responses to transcutaneous SCS than to epidural SCS with either cathode location (transcutaneous SCS vs. epidural SCS with rostral cathode site, P = .006; transcutaneous SCS vs. epidural SCS with caudal cathode site, P = .020), but no statistical difference between the amplitudes of TS responses to epidural SCS with the two different cathode sites tested (P = .856). Mean latencies, peak-to-peak amplitudes, and relative response thresholds are summarized in the S1–S4 Tables.

**Fig 4:**
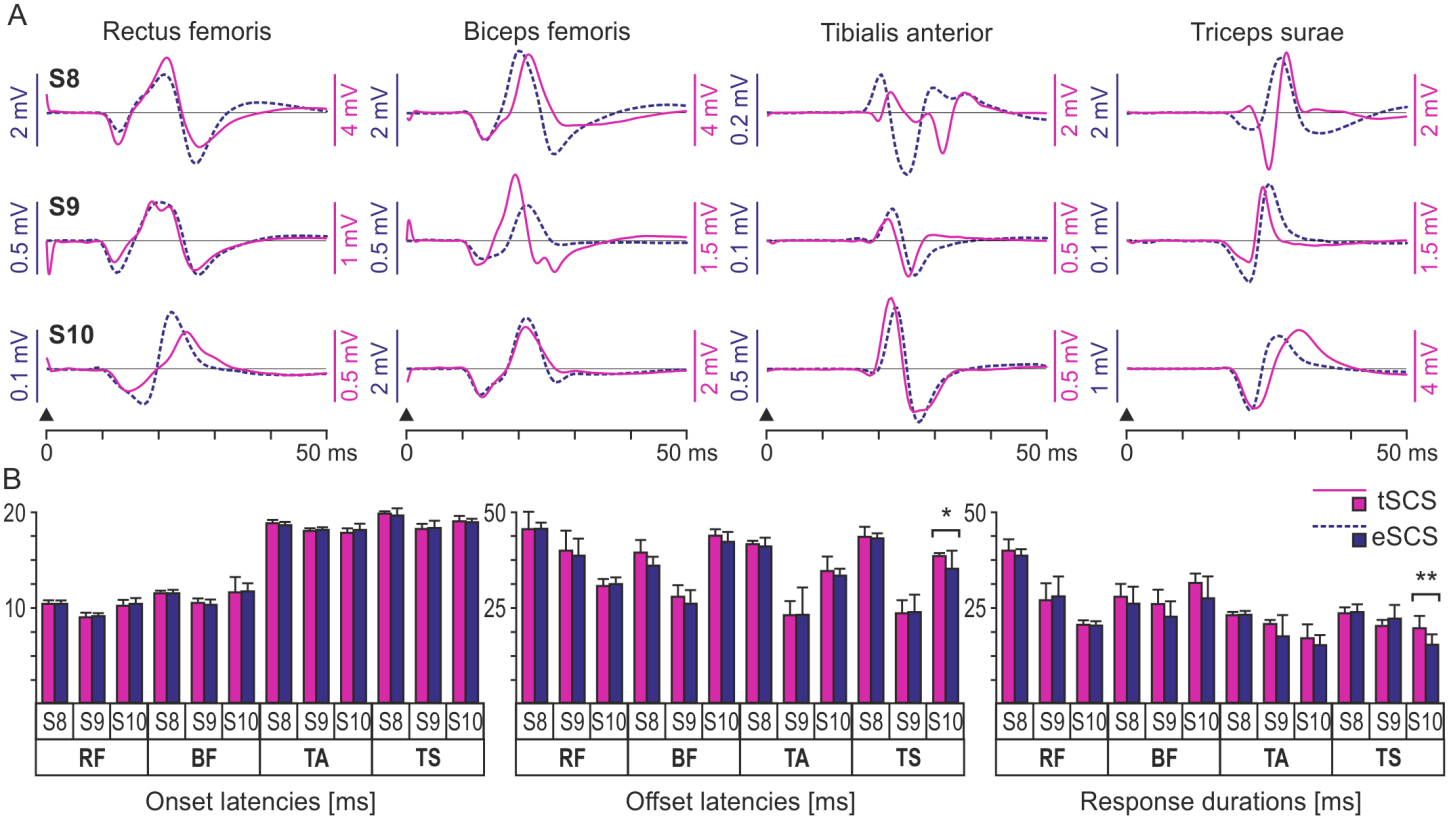
EMG characteristics of responses to transcutaneous and epidural SCS obtained within same individuals. (A) The evoked responses to transcutaneous (tSCS; solid, purple lines) and epidural SCS (eSCS; dashed, blue lines) in rectus femoris (RF), biceps femoris (BF), tibialis anterior (TA), and triceps surae muscle group (TS) appeared in the EMG largely as bi- or triphasic waveforms, with near-identical shapes for both stimulation methods for most muscles. Shown are mean waveforms of the respective evoked potentials after stimulus-triggered averaging; responses in each row are derived from one lower limb each of subjects (S) 8–10. Black arrowheads indicate the respective effective phases of the stimulation pulses. (B) Individual mean onset latencies, offset latencies, and EMG potential durations of the responses to transcutaneous (purple bars) and epidural SCS (blue bars). Error bars indicate SD, asterisks significant differences (*, P < .05; **, P < .01).

Responses to transcutaneous as well as epidural SCS with near-identical EMG waveforms for a given muscle group were obtained in subjects 8, 9, and 10 who were tested with both techniques at different time points (Fig 4A). Individually, no statistical differences were found in the onset latencies (Fig 4B) of the responses of a given muscle group to either stimulation method (Student’s t-test; subject 8, RF, P = .668; BF, P = .066; TA, P = .110; and TS, P = .079; subject 9, RF, P = .257; BF, P = .068; TA, P = .557; and TS, P = .748; subject 10, RF, P = .590; BF, P = .875; TA, P = .137; and TS, P = .724). No differences were detected in the offset latencies (subject 8, RF, P = .838; BF, P = .061; TA, P = .069; and TS, P = .382; subject 9, RF, P = .601; BF, P = .060; TA, P = .105; and TS, P = .064; subject 10, RF, P = .379; BF, P = .052; and TA, P = .191) and response durations (subject 8, RF, P = .428; BF, P = .111; TA, P = .642; and TS, P = .356; subject 9, RF, P = .269; BF, P = .062; TA, P = .085; and TS, P = .056; subject 10, RF, P = .734; BF, P = .067; and TA, P = .055) with the exception of those of the TS responses of subject 10 (offset latencies, P = .029; response durations, P < .001).

## Discussion

Transcutaneous and epidural stimulation of the lumbar spinal cord in individuals with SCI lying supine elicited twitch-like contractions bilaterally in multiple lower-limb muscle groups that were mostly recorded as bi- or triphasic EMG potentials with characteristically short onset latencies. Specifically, responses with EMG amplitudes ≥ 100 μV were evoked by epidural SCS in all muscles studied, and in 77 out of 80 muscles (96.3%) by transcutaneous SCS. In retrospect to subject 7’s recordings, neurographic examination confirmed secondary peripheral nerve lesions, which likely accounted for the lack of responses to transcutaneous SCS in her left TA and TS. The absence of the response in one TA in subject 8 could be explained by the limited stimulation amplitudes applicable to this participant, who reported discomfort below the stimulating surface electrodes with further increases in intensity. Two stimulation pulses applied in close succession demonstrated the depression of the responses to the second stimulus across all muscles for both stimulation techniques, hence corroborating the reflex origin of the evoked responses. There were remarkable similarities between the evoked potentials to epidural and transcutaneous SCS, reflected by highly congruent EMG waveforms and the fact that only 3 out of 16 parameters evaluated (i.e., onset and offset latencies, response durations, peak-to-peak amplitudes, 4 muscles each) showed statistical differences between the two stimulation methods. Particularly, single-pulse transcutaneous and 2-Hz epidural SCS applied with the respective common threshold intensities evoked EMG potentials that attained the same peak-to-peak amplitudes in RF, BF, and TA, whereas the responses of TS to epidural SCS had significantly smaller amplitudes than those evoked by transcutaneous SCS. For a given muscle group, the EMG potentials evoked by either stimulation technique had the same onset latencies, offsets, and response durations across subjects, expect for a shorter onset latency of the responses in TS and a shorter offset latency in BF in case of epidural SCS. The EMG potentials recorded in the three individuals tested with both techniques at different time points had very similar—in some muscles near-identical—waveforms, and no differences were found for their onset and offset latencies or response durations, except for the offsets and durations of the TS responses recorded in one of the subjects.

### Biophysical principles involved in transcutaneous and epidural SCS

The current flow and electric potential distribution generated by transcutaneous and epidural SCS are very dissimilar [27,28,41,59,60]. In epidural stimulation, 80–90% of the ionic current flows between the active electrodes through the cerebrospinal fluid [28,59,60], in which the posterior roots are immersed and which has a considerably higher electrical conductivity than the other anatomical structures close to the electrodes. The electric field generated by epidural stimulation is rather focused, partially depending on the spacing between the selected cathode and anode, permitting a higher segmental selectivity (*cf*. Fig 3A) while also limiting the recruitment of more distant neural structures [27,28]. In transcutaneous SCS, the current flow is strongly influenced by the electrical properties and the numerous conductivity boundaries of the body tissues between the paravertebral and abdominal surface electrodes. Directly below the skin electrodes, relatively high current densities develop [41], causing brief contractions of the paraspinal and abdominal muscles through neuromuscular stimulation (when stimulus amplitude is adequate), and likely the stimulation of superficially located cutaneous fibers as well [61]. Deeper in the body, most of the current flows around the spine, yet some current crosses the spine, mainly via the soft tissues that have considerably higher electrical conductivities than the vertebral bones [28,42]. Computer simulations estimated that about 8% of the overall current flows through the small volume of the dural sac filled with cerebrospinal fluid, where relatively high current densities are produced [28]. The distance between the paravertebral and abdominal electrodes and their dimensions, together with the highly anisotropic volume conductor in-between, cause the electrical field generated to be non-focused and relatively widespread [62,63].

In spite of these differences, modelling studies found that, for both stimulation techniques, large-diameter proprioceptive sensory fibers within the posterior rootlets/roots have the lowest thresholds of all neural structures within the vertebral canal [27,28,42]. In general, the myelinated axon is the most excitable substructure of a neuron in case of externally applied electrical stimulation [64]. Low excitation thresholds of an axon require sudden changes of the electric potential generated along its course and across short distances covering neighboring nodes of Ranvier. More precisely, the strongest depolarization is produced at the site where the second-order spatial derivative of the electric potential along the axon assumes its maximum value [64,65]. In case of epidural SCS, such peak values are generated at the fibers within the longitudinal portions of the posterior rootlets/roots in close vicinity to the stimulating cathode [27,28,66]. Another region of strong depolarization within the cathodic field is at the posterior rootlet-spinal cord junction of the sensory fibers, caused by the sudden voltage drop at the electrical conductivity boundary between the cerebrospinal fluid and the spinal cord, and by the change in spatial orientation of the fibers—from longitudinal to transverse—upon their entrance into the spinal cord [27,28].

Even within the widespread electrical field generated by transcutaneous SCS, computer simulations suggested that these anatomical discontinuities generate localized low-threshold sites along the afferent fibers within the posterior rootlets at their entries into the spinal cord [28,42]. As for motoneurons’ axons, opposite effects are generated at their exits from the spinal cord into the anterior rootlets, since they pass an interface from a low- to a better-conductive medium, and change their course from transverse to longitudinal, resulting in increased thresholds within a cathodic field [28]. Further, since the unfocused electrical field generated transcutaneously does not result in adequate changes of the potential distribution along the longitudinal pathways of the posterior and anterior roots within the dural sac, stimulation of the fibers at these straight sections is highly unlikely [42]. Modelling studies suggested additional low-threshold sites for transcutaneous stimulation of both afferent and efferent fibers in the region of the exits of the posterior and anterior roots from the dural sac, through the epidural fat into the intervertebral foramen [28,41,42]. Yet, in the present study, stimulation at these sites is less likely, because the L2–L5 roots exit the vertebral canal at their respective foramina several vertebral levels distal to the position of the paravertebral electrodes.

It should be stated that both stimulation methods are applied at higher frequencies than those analyzed here when used as neuromodulation interventions. Yet, the biophysical principles determining the directly, electrically stimulated neural structures elaborated above are unaffected by stimulation frequencies within the ranges normally employed in clinical studies (≤ 100 Hz) [13–16,18,21,47–49,51–53,24]. Each stimulation pulse within a series at such frequencies will still electrically activate the same neural input structures to the spinal cord as stimulation with single pulses, but will lead to the generation of action potentials at respectively higher rates. Subsequently, these differences in the afferent input frequencies could lead to different central spinal effects [18,19,33,67]. Interestingly, the stimulation frequency ranges that have been used to either control spinal spasticity (50–100 Hz) [13,47,48] or facilitate locomotor function (20–50 Hz) [15,21,51,24] in individuals with SCI have been the same for transcutaneous and epidural SCS.

### The EMG potentials evoked by transcutaneous and epidural SCS

Responses to low-frequency epidural [18,29,32,33] and transcutaneous [40,45,55] SCS are recorded as stimulus-triggered EMG potentials with latencies and waveforms that are characteristic for a given muscle group. We applied single-pulse transcutaneous SCS with an interval of 15 s between successive stimuli to minimize potential discomfort due to the concomitant neuromuscular stimulation of the paraspinal and abdominal muscles underneath the skin electrodes, as well as to rule out influences on the evoked responses caused by the repeated input to the lumbar spinal circuitry. In the case of epidural SCS, we analyzed responses to repetitive stimulation at the lowest programmable stimulation frequency of 2 Hz, since single-pulse application is not possible with commercial implantable pulse generators. It was recently shown that PRM reflexes elicited by trains of 2-Hz stimulation delivered epidurally in individuals with SCI (lying relaxed in a supine position) are not subject to amplitude depression or modulation of other EMG characteristics, probably because at this frequency, changes in synaptic transmission and motoneuronal responsiveness with repetitive multi-root activation largely even each other out [33].

The onset latencies of PRM reflexes are essentially defined by the site of afferent fiber depolarization, the segmental, central conduction time, the number of synapses intercalated in the reflex arc, the length of the efferent pathway to the recorded lower-limb muscle, the conduction velocities of the involved afferent and efferent fibers, and further, the delays at the neuromuscular junctions and conduction along the muscle fibers towards the recording electrodes [18,33,40,43]. The attainable peak-to-peak amplitudes and waveforms of the reflex EMG potentials depend on the number of afferent fibers recruited, on pre- and postsynaptic processes that can modulate, at spinal level, the afferent input arriving at the motoneurons as well as the motoneuronal excitability, the resultant recruitment of the motoneuron pools, and the weighted summation of the electrical activity of the muscle fibers within the detection volume of the surface-EMG electrodes [68]. Subject-specific characteristics will also influence the onset latencies and the attainable amplitudes of the evoked potentials, primarily subject height, time since injury, and age, which did not differ between the groups tested with transcutaneous and epidural SCS. In the transcutaneous group, there were yet two outliers in age (subjects 1 and 4, Table 1), who would have been most affected by age-related variations in reflex latency [69], decline in muscle mass [70], changes in the intrinsic properties of muscle contractile elements and metabolic characteristic [71,72] as well as alterations in the neuromuscular system [73]. Indeed, onset latencies derived from these two subjects were among the longest within the group tested with transcutaneous SCS, but this variation did not affect a single muscle specifically (see S2 Table). The peak-to-peak amplitudes of the responses were within the group ranges.

Apart from these physiological aspects, there are several other factors that can influence the shape and amplitude of EMG potentials, including, e.g., the specific surface-EMG electrode and amplifier properties, the quality of the contact between the electrodes and the skin, the specific properties of subcutaneous tissues between the active muscle area and the electrodes, the inter-electrode distance, and the location of the EMG electrodes relative to the innervation zones of the muscle fibers [74–76]. Amplitude normalization of the studied responses to the EMG activity during maximum voluntary contractions or to the maximum M wave evoked by supramaximal stimulation of mixed peripheral nerves [68] could take these factors into account. Yet, both techniques were not applicable in the present study, due to the motor impairments of the participants and because not all nerves in the periphery associated with the muscles studied are accessible to or can be completely recruited by supramaximal electrical stimulation. Nevertheless, absolute peak-to-peak amplitudes and attainable sizes are key characteristics of evoked responses. For instance, the values reported here for TS are within the ranges of those of monosynaptic reflexes, while they are higher by up to one order of magnitude than those of motor evoked potentials elicited by transcranial magnetic stimulation [68,77–79].

All facts considered, the EMG potentials to transcutaneous and epidural SCS with their near-identical temporal EMG characteristics, waveforms, and attainable peak-to-peak amplitudes provide ample evidence that the responses to either technique were PRM reflexes and that both techniques indeed activated the same neural input structures to the spinal cord—or at least a common subset—at similar sites. Based on *(i)* the biophysical considerations discussed above, *(ii)* the reflex nature of the evoked responses, and *(iii)* the fact that the largest-diameter axons have the lowest activation thresholds in case of externally applied electrical stimulation [80], these structures included, in the first instance, large-diameter group Ia proprioceptive posterior-root afferent fibers that originate from muscle spindle primary endings in the legs [27,28,42]. The reflex responses were then primarily caused via monosynaptic homonymous Ia projections to motoneurons, based on the particular efficacy of these excitatory connections [81]. In addition, because each pulse with the intensities analyzed here activated several posterior roots containing proprioceptive afferent fibers from multiple lower limb muscles, heteronymous Ia excitation [82,83] likely contributed to the reflex responses as well [57]. Group Ib afferents from Golgi tendon organs, which have fiber sizes that overlap with those of group Ia, were likely activated similarly effectively. Their effect upon the motoneurons, delayed only by the time required to cross a non-reciprocal group I inhibitory interneuron, could be to curtail the size of the reflex output at higher stimulation intensities [68,69]. Further, the recruitment of additional, medium-diameter fibers can be also assumed since both stimulation techniques normally induce paraesthesias, i.e., tingling sensations, when applied at higher frequencies (e.g., 50 Hz), even with stimulation intensities lower than those analyzed here (common threshold) [43,47,84]. The generation of paraesthesias by SCS is associated with the activation of A-beta fibers of afferents from mechanoreceptors of the skin that correspond to group II according to the Lloyd-Hunt classification of afferent fibers [60,84,85]. The contribution of group II fibers from muscle spindle secondary endings or cutaneous mechanoreceptors to the evoked potentials as analyzed here is less clear. Experiments using epidural SCS in rats suggested that group II stimulation could be the cause of a ‘late potential’ recorded in the EMG as a separate oligo-/polysynaptic response immediately following the monosynaptic reflex [35,36]. Such EMG responses with clearly separate early and late potentials were not detected here within the 50 ms time windows of analysis. Comparison of the EMG potentials of the classical, largely monosynaptic H reflex evoked in the tibial nerve and the PRM reflex in the same muscle evoked by transcutaneous SCS in individuals with intact nervous system showed that the respective EMG waveforms were nearly identical when superimposed (after correcting for the difference in the latency times of about 11.5 ms) [40,79]. The EMG waveforms of the TS responses recorded in our study are also reminiscent of that of the H reflex. However, some evoked potentials detected here (e.g., TA of subject 8 in Fig 4) might be interpreted as a superposition of an early and a slightly delayed EMG response. We thus suggest that the evoked responses to single-pulse transcutaneous or low-frequency epidural SCS mainly reflect homonymous monosynaptic excitation with additional heteronymous Ia excitatory influence following the multi-root stimulation, and some examples may incorporate oligosynaptic contributions from group II afferents [36].

Despite their overwhelming similarities, we detected differences in a few EMG parameters of the responses to epidural and transcutaneous SCS. The onset latencies of the TS responses were longer by 1.6 ms on average in the transcutaneous SCS group compared to those evoked in the epidural SCS group. These results are also in accordance with previous work reporting comparable onset latencies of the responses to transcutaneous [40] as well as epidural SCS [33]. Notably, the PRM reflexes recorded from TS are initiated in the most caudal roots of the muscles studied here, including the S1 and S2 roots, which enter caudally into the sacral canal. The electric potential distribution generated by transcutaneous SCS at the transition from the fifth lumbar vertebra to the comparatively massive and less conductive bony structure of the sacrum has not been studied yet, and the existence of a not yet described low-threshold site for these roots created at the transition from the vertebral to the sacral canal cannot be completely ruled out. Hence, one potential explanation for the longer onset latencies of the TS responses is that transcutaneous SCS depolarized the S1 and S2 roots at more distant sites in some individuals, thereby increasing the distance within the afferent limb of the reflex arc to be travelled by the electrically evoked action potentials. Yet, we would not expect that afferent fibers were regularly recruited within the cauda equina region in the present study since previous work using paravertebral stimulating electrodes placed over the L4 and L5 spinous processes showed that such caudal stimulation would further delay the respective onset latencies of the TS responses to 21.3 ± 1.1 ms (from 19.7 ± 1.1 ms with stimulation electrodes over T11 and T12) [40]. Even in case of such distant activation, the driving afferent input arriving at the spinal cord would be still very similar to that of epidural SCS. Another potential source for the different onset latencies could be systematic differences in the specific placement of the surface-EMG electrodes over the TS [75], since the EMG electrodes in the epidural SCS group were placed, in most cases, by a team of two lab technicians, and those in the transcutaneous SCS group were placed by two research assistants. While EMG electrode locations were consistent for RF, BF, and TA (see Methods), there were small differences in EMG-electrode placement over the calf muscle group, either in the midline, at the end of the two heads of the gastrocnemius muscle (epidural group), or slightly more distally (≤ 2 cm) to also partially cover the soleus muscle (transcutaneous group). This difference may have led to the recording of different contributions of the TS muscle group to the EMG potentials, impacting the initial phase of the EMG waveform and thus potentially also the identification of the onset latency. This factor alone, however, cannot account for the observed difference of 1.6 ms. Alrowayeh et al. [86] recorded H reflexes to tibial nerve stimulation with surface electrodes explicitly placed over the soleus, lateral gastrocnemius and medial gastrocnemius muscles. Even with these distinct electrode sites, with larger distances between electrodes than between the epidural and transcutaneous groups in our study, the latency difference between gastrocnemius and soleus responses was 0.9 ms. A combination of some of the factors discussed above could have led to the observed difference in the TS onset latencies. Interestingly, no such difference was present in the three individuals who were tested by both techniques. Differences in the decaying phases of the evoked EMG potentials accounted for the shorter offset latencies of the BF responses to epidural SCS. A potential explanation for the smaller peak-to-peak amplitudes of the TS responses to epidural than to transcutaneous SCS could be that, with the bias of the epidural electrode placements over the (upper) lumbar spinal cord [13], together with the more focal field produced—and thus the limited stimulation range—the S1 and S2 roots were not sufficiently recruited at the common threshold intensity. Furthermore, seven of the ten individuals tested with epidural stimulation had a clinically classified motor-complete SCI (6 AIS A, 1 AIS B; *cf*. Table 1), whereas nine of the patients in the transcutaneous SCS group had motor-incomplete lesions (4 AIS C, 5 AIS D). Previous studies of the soleus H reflex revealed significantly smaller M_max_ amplitudes in individuals with complete compared to incomplete SCI [87]. As there was no correlation of the H_max_-to-M_max_ ratio with the severity of SCI [87,88], also the sizes of H_max_ must have been smaller in case of motor-complete lesions. Along this line, earlier studies showed greater soleus stretch reflexes [88] as well as responses to Achilles’ tendon taps [89] in motor-incomplete vs. complete SCI that were attributed to differences in long-tract sparing and secondary mechanical adaptations in the muscle and tendon complex associated with different SCI severities [87–89]. The differences in lesion severity between the transcutaneous and the epidural SCS group may have also contributed to the differences in post-activation depression found in BF [87].

### Methodological differences between transcutaneous and epidural SCS

While the present results support the principle conformity of the direct effects of both stimulation techniques on posterior-root fibers when applied in a single-pulse mode or at a low frequency and with common threshold intensity, there are still methodological differences that may impact the respective fields of application and therapeutic efficacy. As stated above, epidural SCS produces a more localized electrical field that results in a higher segmental selectivity of the recruited posterior roots [18,29,30,32]. This selectivity was also reflected by the present data that showed differences in the thresholds of RF and TS responses, i.e., the two muscle groups studied that have separate segmental innervations (L2–L4 and L5–S2, respectively) [90,91], depending on the rostrocaudal position of the cathode. Using multi-electrode paddle leads comprised of several rostrocaudally and mediolaterally arranged contacts [15,24] as well as innovative algorithms for closed-loop, real-time control of task- and phase-specific stimulation of specific groups of posterior roots, epidural SCS hence appears as a potent tool to augment, or induce, functional movement after severe SCI, likely also leading to superior outcomes in gait therapy [92,93]. The comparatively distant and unfocused electrical field produced by transcutaneous SCS [62,63] with the electrode set-up used here did not demonstrate a segmental selectivity of posterior-root stimulation. Alternative electrode designs and their specific placement may allow for some rostrocaudal selectivity [94], while the right-left selectivity of posterior root stimulation remains to be demonstrated. Furthermore, because of the use of surface electrodes and body-position dependent variations in the stimulation conditions [55], transcutaneous SCS cannot be applied chronically. The particular strength of transcutaneous SCS lies, first and foremost, in its simple and noninvasive nature, and it may rather find applications where the relatively uniform coverage of several spinal cord segments bilaterally—by a tonic mode of stimulation—is required, e.g., in the treatment of diffuse forms of lower-limb spasticity [47,48], or for increasing the general central state of excitability of lumbar spinal cord circuits to enhance the outcome of locomotor therapies [26,49,51].

## Conclusions

The near identical EMG potentials evoked by epidural and transcutaneous lumbar SCS at comparable relative intensities acquired from several key muscle groups of the lower extremities highly suggest that both techniques activate common neuronal structures. Together with the known biophysical principles involved and the reflex nature of the responses, the similarity of the evoked EMG potentials complements the evidence that both methods stimulate afferent fibers within the L2 to S2 posterior rootlets or roots. These include, in the first instance, proprioceptive afferent fibers that originate from muscle spindle primary endings in the legs. This finding is not only important for future applications of tonic transcutaneous SCS in novel neuromodulation approaches, but also further adds to the mechanistic and conceptual framework of epidural SCS. The present results hence substantially contribute to further advancing the understanding of the basic mechanisms underlying electrical stimulation of the human lumbar spinal cord.

## Supporting information

S1 Fig Supplementary experiments confirming the effective phase of the biphasic rectangular stimulation pulses applied through transcutaneous lumbar SCS. Biphasic, symmetric rectangular pulses were applied such that the paraspinal electrodes acted as the anode for the first and the cathode for the second phase of the pulse with reference to the abdominal electrodes. Three single stimuli were applied with pulse widths of 0.5 ms to 5 ms per phase as indicated. Stimulation artifacts (stim. art.) were detected by the EMG electrodes over biceps femoris (BF), the EMG site closest to the location of the paravertebral stimulation electrodes (grey EMG traces). (A) BF and triceps surae (TS) traces aligned (green line) with reference to the onset of the stimulation pulses. Onset latencies of TS responses calculated from the onset of the stimulation artifact were 20.6 ± 0.3 ms (0.5 ms pulse width per phase), 22.6 ± 0.2 ms (2.5 ms pulse width per phase), and 25.1 ± 0.1 ms (5 ms pulse width per phase). This shift in latency detection with increasing pulse widths is indicated by the dotted line. (B) Responses aligned (green line) with reference to the transition (highlighted in red color) between the two phases of the biphasic pulses, when the polarity of the paraspinal electrodes changed from anode to cathode with respect to the abdominal electrodes. Onset latencies of TS responses calculated from this phase transition were 20.0 ± 0.2 ms (0.5 ms pulse width per phase), 20.1 ± 0.2 ms (2.5 ms pulse width per phase), and 20.1 ± 0.2 ms (5 ms pulse width per phase). These observations confirm that the evoked responses were initiated by the abrupt change of polarity of the biphasic stimulation pulses. Stimulation amplitudes were adapted to obtain TS responses of similar sizes for the different pulse widths; subject 8.
(TIF)

S1 Table Average latencies and peak-to-peak amplitudes (± SE) of responses to single-pulse transcutaneous SCS as well as 2-Hz epidural SCS applied with respective common threshold intensities. (PDF)

S2 Table Individual latencies and peak-to-peak amplitudes (mean ± SD) of responses to single-pulse transcutaneous SCS applied with respective common threshold intensities. (PDF)

S3 Table Individual latencies and peak-to-peak amplitudes (mean ± SD) of responses to 2-Hz epidural SCS applied with respective common threshold intensities. (PDF)

S4 Table Threshold intensities to evoked muscle twitches in the various leg muscles normalized to the respective individually identified lowest response threshold. (PDF)
