# Supplementary figures and images for: Common neural structures activated by epidural and transcutaneous lumbar spinal cord stimulation: Elicitation of posterior root-muscle reflexes
Source: PLoS One. 2018 Jan 30;13(1):e0192013. doi: 10.1371/journal.pone.0192013 (PMC5790266; doi:10.1371/journal.pone.0192013)

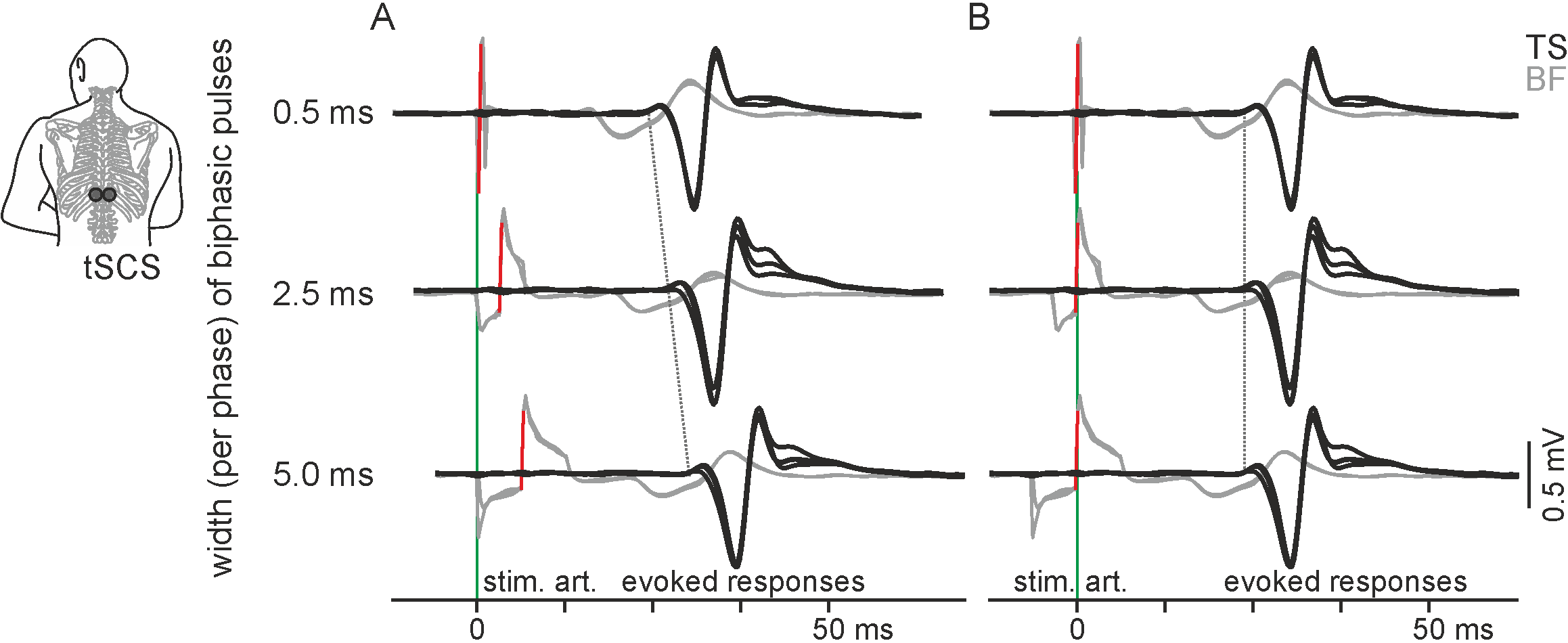

Supplement: S1 Fig — Biphasic, symmetric rectangular pulses were applied such that the paraspinal electrodes acted as the anode for the first and the cathode for the second phase of the pulse with reference to the abdominal electrodes. Three single stimuli were applied with pulse widths of 0.5 ms to 5 ms per phase as indicated. Stimulation artifacts (stim. art.) were detected by the EMG electrodes over biceps femoris (BF), the EMG site closest to the location of the paravertebral stimulation electrodes (grey EMG traces). (A) BF and triceps surae (TS) traces aligned (green line) with reference to the onset of the stimulation pulses. Onset latencies of TS responses calculated from the onset of the stimulation artifact were 20.6 ± 0.3 ms (0.5 ms pulse width per phase), 22.6 ± 0.2 ms (2.5 ms pulse width per phase), and 25.1 ± 0.1 ms (5 ms pulse width per phase). This shift in latency detection with increasing pulse widths is indicated by the dotted line. (B) Responses aligned (green line) with reference to the transition (highlighted in red color) between the two phases of the biphasic pulses, when the polarity of the paraspinal electrodes changed from anode to cathode with respect to the abdominal electrodes. Onset latencies of TS responses calculated from this phase transition were 20.0 ± 0.2 ms (0.5 ms pulse width per phase), 20.1 ± 0.2 ms (2.5 ms pulse width per phase), and 20.1 ± 0.2 ms (5 ms pulse width per phase). These observations confirm that the evoked responses were initiated by the abrupt change of polarity of the biphasic stimulation pulses. Stimulation amplitudes were adapted to obtain TS responses of similar sizes for the different pulse widths; subject 8. (TIF) [file pone.0192013.s001.tif]
