# Supplementary material for: Common neural structures activated by epidural and transcutaneous lumbar spinal cord stimulation: Elicitation of posterior root-muscle reflexes
Source: PLoS One. 2018 Jan 30;13(1):e0192013. doi: 10.1371/journal.pone.0192013 (PMC5790266; doi:10.1371/journal.pone.0192013)
Supplement: S1 Table — (PDF) [file pone.0192013.s002.pdf]

S1 Table. Average latencies and peak-to-peak amplitudes (± SE) of responses to single-pulse transcutaneous as well as 2-Hz epidural spinal cord stimulation applied with respective common threshold intensities.

	Rectus femoris	Biceps femoris	Tibialis anterior	Triceps surae
Onset latencies (ms)				
tSCS	9.8 ± 0.4	11.0 ± 0.3	18.7 ± 0.4	19.8 ± 0.4
eSCS	9.6 ± 0.3	10.9 ± 0.2	18.5 ± 0.3	18.2 ± 0.3
Offsets (ms)				
tSCS	33.2 ± 1.6	40.7 ± 0.7	40.5 ± 1.3	37.8 ± 0.9
eSCS	35.9 ± 1.2	37.8 ± 1.1	37.7 ± 1.6	36.2 ± 1.4
Response durations (ms)				
tSCS	23.1 ± 1.4	29.5 ± 0.8	22.0 ± 1.6	18.0 ± 0.8
eSCS	26.3 ± 1.3	27.2 ± 1.1	19.5 ± 1.5	19.0 ± 1.4
Peak-to-peak amplitudes (mV)				
tSCS	1.6 ± 0.4	2.0 ± 0.4	0.8 ± 0.2	3.0 ± 0.5
eSCS, 0–3+	1.6 ± 0.4	1.7 ± 0.3	0.5 ±0.1	1.3 ± 0.3
eSCS, 0+3–	1.0 ± 0.4	1.8 ± 0.3	0.7 ± 0.1	1.5 ± 0.3

eSCS, epidural spinal cord stimulation; tSCS, transcutaneous spinal cord stimulation; 0–3+, most rostral cathode site along the epidural array; 0+3–, most caudal cathode site. Note that for the calculation of onset and offset latencies as well as the response durations, data obtained with both bipolar electrode combinations of epidural SCS were pooled.
