# Supplementary material for: Common neural structures activated by epidural and transcutaneous lumbar spinal cord stimulation: Elicitation of posterior root-muscle reflexes
Source: PLoS One. 2018 Jan 30;13(1):e0192013. doi: 10.1371/journal.pone.0192013 (PMC5790266; doi:10.1371/journal.pone.0192013)
Supplement: S2 Table — (PDF) [file pone.0192013.s003.pdf]

S2 Table. Individual latencies and peak-to-peak amplitudes (mean ± SD) of responses to single-pulse transcutaneous spinal cord stimulation applied with respective common threshold intensities.

Subject	Rectus femoris	Biceps femoris	Tibialis anterior	Triceps surae
Onset latencies (ms)				
1	11.9 ± 0.5	12.3 ± 0.6	19.7 ± 0.7	21.7 ± 0.5
2	10.0 ± 0.2	11.4 ± 0.3	18.6 ± 1.0	20.6 ± 0.8
3	10.1 ± 0.2	11.6 ± 0.1	17.7 ± 1.2	21.6 ± 0.6
4	10.0 ± 0.3	11.5 ± 0.1	21.5 ± 0.6	20.0 ± 0.7
5	9.5 ± 0.2	10.1 ± 0.5	19.0 ± 0.6	19.3 ± 0.4
6	9.2 ± 0.7	10.3 ± 0.6	19.1 ± 0.3	18.5 ± 0.1
7	7.4 ± 0.1	9.8 ± 0.1	16.2 ± 0.1	18.6 ± 0.8
8	10.3 ± 0.2	11.5 ± 0.1	18.9 ± 0.1	20.3 ± 0.8
9	9.6 ± 0.9	10.3 ± 0.3	18.2 ± 0.9	18.1 ± 0.4
10	10.1 ± 0.5	11.5 ± 1.2	17.7 ± 0.4	18.9 ± 0.2
Offsets (ms)				
1	38.1 ± 4.7	37.8 ± 5.3	44.8 ± 4.9	40.2 ± 5.2
2	28.6 ± 2.6	38.4 ± 3.7	44.9 ± 4.8	37.1 ± 5.5
3	33.6 ± 0.6	41.6 ± 3.2	45.9 ± 3.7	36.7 ± 2.1
4	37.5 ± 5.1	43.2 ± 5.5	36.8 ± 3.5	40.3 ± 4.2
5	33.4 ± 1.2	39.7 ± 4.6	40.5 ± 3.9	36.0 ± 4.7
6	26.7 ± 2.8	42.8 ± 4.1	34.5 ± 4.7	37.6 ± 3.8
7	26.9 ± 4.0	39.2 ± 4.3	41.9 ± 1.6	32.2 ± 3.6
8	41.0 ± 4.5	39.1 ± 3.0	41.3 ± 0.6	42.1 ± 4.8
9	35.4 ± 3.6	42.1 ± 4.0	38.7 ± 3.4	37.3 ± 3.0
10	30.4 ± 1.5	43.5 ± 1.4	35.6 ± 3.8	38.2 ± 0.4
Response durations (ms)				
1	25.8 ± 4.0	25.6 ± 4.9	27.4 ± 6.1	18.5 ± 5.4
2	18.6 ± 2.7	27.1 ± 3.7	26.3 ± 4.1	16.5 ± 5.1
3	23.5 ± 0.7	30.0 ± 3.1	28.7 ± 4.2	14.9 ± 1.7
4	27.5 ± 5.9	31.7 ± 5.5	14.5 ± 4.5	20.5 ± 5.2
5	23.9 ± 1.1	29.6 ± 5.3	21.5 ± 3.8	16.7 ± 5.1
6	15.8 ± 2.5	32.6 ± 4.5	15.4 ± 4.9	19.1 ± 3.7
7	19.5 ± 4.0	26.5 ± 4.5	25.8 ± 1.6	13.6 ± 3.3
8	30.7 ± 4.7	27.6 ± 3.0	22.4 ± 0.5	21.2 ± 4.9
9	25.8 ± 3.5	31.8 ± 5.9	20.5 ± 4.0	19.2 ± 3.1
10	20.3 ± 1.0	32.0 ± 2.4	17.9 ± 3.8	19.4 ± 0.4
Peak-to-peak amplitudes (µV)				
1	1451.0 ± 388.6	2688.7 ± 262.8	830.3 ± 174.4	3541.9 ± 461.3
2	688.2 ± 222.6	1336.2 ± 209.9	322.6 ± 81.1	1538.7 ± 271.1
3	1510.6 ± 511.7	3640.9 ± 682.4	160.8 ± 27.9	2381.2 ± 769.1
4	2673.6 ± 232.4	1823.9 ± 378.5	970.9 ± 452.0	1978.6 ± 594.1
5	1898.1 ± 522.5	1078.2 ± 195.5	535.3 ± 115.2	4358.9 ± 1040.8

6	386.7 ± 230.3	595.6 ± 197.5	1407.4 ± 379.0	4202.7 ± 769.3
7	636.0 ± 233.2	1053.9 ± 297.0	333.8 ± 29.9	252.2 ± 61.4
8	4372.4 ± 512.0	4634.7 ± 113.1	1796.9 ± 120.1	2801.7 ± 726.0
9	1544.1 ± 911.9	1867.5 ± 387.1	970.8 ± 514.9	4279.5 ± 783.5
10	523.0 ± 140.1	1453.4 ± 517.1	899.5 ± 154.6	4866.4 ± 626.4
