# Supplementary material for: Common neural structures activated by epidural and transcutaneous lumbar spinal cord stimulation: Elicitation of posterior root-muscle reflexes
Source: PLoS One. 2018 Jan 30;13(1):e0192013. doi: 10.1371/journal.pone.0192013 (PMC5790266; doi:10.1371/journal.pone.0192013)
Supplement: S3 Table — (PDF) [file pone.0192013.s004.pdf]

S3 Table. Individual latencies and peak-to-peak amplitudes (mean ± SD) of responses to 2-Hz epidural spinal cord stimulation applied with respective common threshold intensities.

Subject	Rectus femoris	Biceps femoris	Tibialis anterior	Triceps surae
Onset latencies (ms)				
8	10.9 ± 0.9	10.9 ± 0.4	17.4 ± 0.4	18.9 ± 1.1
9	9.0 ± 0.3	10.2 ± 0.4	18.1 ± 0.3	18.2 ± 0.6
10	10.6 ± 0.5	11.0 ± 0.9	19.0 ± 0.9	18.4 ± 0.5
11	9.7 ± 0.3	11.7 ± 1.2	18.7 ± 0.5	19.7 ± 0.2
12	10.0 ± 0.1	10.6 ± 0.3	18.5 ± 0.5	17.2 ± 0.5
13	9.0 ± 0.6	10.6 ± 0.9	18.2 ± 0.7	17.0 ± 0.5
14	9.0 ± 0.3	9.9 ± 0.3	18.8 ± 0.9	17.9 ± 0.6
15	9.9 ± 0.4	11.2 ± 0.8	19.8 ± 0.3	19.2 ± 0.3
16	9.8 ± 0.3	11.8 ± 0.4	19.4 ± 0.7	18.0 ± 0.4
17	8.1 ± 1.0	10.6 ± 0.2	17.3 ± 0.4	17.0 ± 0.8
Offsets (ms)				
8	37.3 ± 1.4	36.5 ± 3.6	40.7 ± 2.1	42.5 ± 1.5
9	36.0 ± 5.0	32.7 ± 4.5	34.5 ± 4.9	30.3 ± 4.2
10	38.1 ± 4.7	37.4 ± 4.9	31.4 ± 2.3	34.9 ± 4.3
11	30.3 ± 3.5	34.7 ± 5.7	43.5 ± 3.5	28.8 ± 1.5
12	28.4 ± 0.8	41.9 ± 2.2	35.1 ± 3.0	37.4 ± 3.4
13	36.1 ± 3.3	39.8 ± 2.8	30.7 ± 2.7	33.2 ± 2.4
14	39.3 ± 2.1	34.6 ± 4.4	39.1 ± 4.9	41.4 ± 5.2
15	34.5 ± 2.8	36.5 ± 2.2	44.8 ± 1.3	39.6 ± 2.9
16	38.7 ± 5.3	42.7 ± 0.9	42.2 ± 3.3	36.3 ± 3.7
17	39.9 ± 4.4	40.7 ± 5.2	35.1 ± 4.3	37.6 ± 4.0
Response durations (ms)				
8	26.5 ± 1.4	25.8 ± 4.1	24.1 ± 3.3	23.6 ± 2.1
9	27.0 ± 4.9	23.1 ± 5.5	17.3 ± 5.9	21.9 ± 5.4
10	27.5 ± 5.3	26.4 ± 5.6	12.7 ± 3.2	16.6 ± 4.7
11	20.5 ± 3.6	23.1 ± 5.7	25.8 ± 5.9	9.1 ± 2.8
12	18.4 ± 0.7	31.3 ± 2.4	16.7 ± 2.7	20.2 ± 3.0
13	27.1 ± 3.5	29.1 ± 3.2	12.5 ± 2.7	16.2 ± 2.7
14	30.4 ± 2.0	24.7 ± 4.3	20.7 ± 6.0	23.4 ± 5.6
15	24.7 ± 2.8	25.3 ± 2.7	25.0 ± 1.2	20.5 ± 3.0
16	28.9 ± 5.0	30.8 ± 0.9	22.8 ± 3.2	18.3 ± 3.8
17	31.8 ± 4.3	32.6 ± 5.1	17.3 ± 4.6	19.8 ± 5.2
Peak-to-peak amplitudes (µV), rostral cathode site along the epidural array (0–3+)				
8	2953.3 ± 322.2	1646.6 ± 98.5	138.3 ± 95.9	910.1 ± 228.9
9	872.2 ± 193.8	476.1 ± 200.5	95.7 ± 13.1	112.4 ± 24.1
10	177.2 ± 42.1	1178.5 ± 222.5	499.0 ± 310.9	558.7 ± 217.2
11	3027.9 ± 606.1	2154.6 ± 311.6	521.2 ± 108.0	1115.9 ± 221.7
12	3471.9 ± 226.5	2872.0 ± 150.5	751.0 ± 193.7	2360.1 ± 470.6
13	922.2 ± 494.7	2462.2 ± 156.4	1210.6 ± 158.7	2634.3 ± 311.5

14	352.9 ± 197.9	467.3 ± 162.9	256.2 ± 88.5	542.9 ± 51.4
15	1885.3 ± 101.7	2017.0 ± 580.2	453.1 ± 177.4	1042.2 ± 420.0
16	1625.2 ± 527.6	2657.0 ± 547.1	1025.1 ± 444.6	2966.9 ± 112.7
17	336.1 ± 26.0	710.6 ± 53.4	213.1 ± 48.1	394.2 ± 92.8
Peak-to-peak amplitudes (μV), caudal cathode site along the epidural array (0+3–)				
8	1032.1 ± 210.2	1952.2 ± 125.6	223.6 ± 105.9	1474.7 ± 231.6
9	106.6 ± 37.2	532.1 ± 319.5	592.8 ± 306.1	526.2 ± 195.7
10	138.8 ± 12.1	1812.0 ± 625.4	641.9 ± 180.7	1312.5 ± 432.2
11	2962.2 ± 77.3	2188.0 ± 189.4	534.3 ± 223.2	552.0 ± 419.3
12	4116.5 ± 76.4	2760.9 ± 278.3	914.0 ± 174.7	2259.1 ± 465.0
13	373.0 ± 216.0	1550.5 ± 266.0	1654.0 ± 337.3	2862.7 ± 147.8
14	326.8 ± 101.0	910.8 ± 236.7	475.6 ± 88.1	2153.8 ± 342.6
15	99.0 ± 17.5	1716.4 ± 276.0	597.6 ± 57.6	1218.2 ± 442.8
16	250.1 ± 92.8	3504.3 ± 351.3	896.3 ± 366.8	2446.1 ± 191.6
17	125.2 ± 23.7	727.8 ± 110.6	493.4 ± 201.2	473.7 ± 127.0
