# Supplementary material for: Common neural structures activated by epidural and transcutaneous lumbar spinal cord stimulation: Elicitation of posterior root-muscle reflexes
Source: PLoS One. 2018 Jan 30;13(1):e0192013. doi: 10.1371/journal.pone.0192013 (PMC5790266; doi:10.1371/journal.pone.0192013)
Supplement: S4 Table — (PDF) [file pone.0192013.s005.pdf]

S4 Table. Threshold intensities to evoke EMG responses with peak-to-peak amplitudes ≥ 100 μV in lower-limb muscles, individually normalized to the lowest threshold across muscles.

Subject	RRF	LRF	RBF	LBF	RTA	LTA	RTS	LTS
Transcutaneous spinal cord stimulation								
1	1.3	1.4	1.0	1.0	1.3	1.0	1.2	1.0
2	1.3	1.1	1.6	1.0	1.8	1.1	1.3	1.2
3	1.3	1.3	1.1	1.0	1.2	1.2	1.1	1.1
4	1.0	1.0	1.0	1.0	2.4	1.9	1.7	1.7
5	1.3	1.2	1.1	1.0	1.1	1.1	1.1	1.1
6	4.3	4.3	1.5	1.5	1.3	1.0	1.1	1.0
7	1.0	1.2	1.0	1.2	1.2	NA	1.5	NA
8	1.3	1.0	1.3	1.3	1.3	NA	2.5	2.5
9	1.3	1.4	1.4	1.0	1.5	1.0	1.5	1.0
10	1.3	1.4	1.2	1.2	1.3	1.3	1.1	1.0
Epidural spinal cord stimulation, most rostral cathode site along the array								
8	1.6	1.0	1.4	1.1	1.6	1.8	2.3	1.6
9	1.0	1.5	1.0	2.0	3.5	3.5	3.5	3.5
10	1.6	1.4	1.6	1.0	1.6	1.0	1.6	1.0
11	1.8	1.0	1.5	1.0	2.5	2.0	2.5	2.0
12	1.0	1.0	1.0	1.0	1.5	1.5	1.5	1.5
13	1.5	1.5	3.0	1.5	2.0	1.0	2.0	1.4
14	1.2	1.0	1.2	1.0	1.2	1.0	1.2	1.1
15	1.0	1.0	1.0	1.0	1.7	1.0	1.7	1.2
16	1.3	1.3	1.0	1.3	1.0	1.3	1.2	1.3
17	1.0	1.0	1.0	1.0	1.3	1.2	1.3	1.2
Epidural spinal cord stimulation, most caudal cathode site along the array								
8	2.1	1.3	1.1	1.0	2.6	1.9	1.6	1.6
9	1.3	1.3	1.3	1.0	1.3	1.0	1.3	1.0
10	3.0	2.0	2.5	1.0	2.5	1.0	2.0	1.0
11	3.0	1.8	2.0	1.0	3.0	1.0	1.3	1.0
12	1.2	1.5	1.0	1.5	1.5	1.5	1.5	1.5
13	4.0	2.0	4.0	2.0	2.0	1.0	1.8	1.0
14	1.3	1.2	1.3	1.0	1.0	1.0	1.0	1.0
15	1.5	1.5	1.5	1.0	1.0	1.0	1.0	1.0
16	1.6	3.0	1.5	2.0	1.5	2.0	1.0	1.5
17	1.1	1.3	1.0	1.0	1.3	1.3	1.0	1.3

EMG, electromyographic; right (R) and left (L) BF, biceps femoris; RF, rectus femoris; TA, tibialis anterior; and TS, triceps surae muscle group.
